# Melatonin preconditioning is an effective strategy for mesenchymal stem cell‐based therapy for kidney disease

**DOI:** 10.1111/jcmm.14769

**Published:** 2019-11-20

**Authors:** Lingfei Zhao, Chenxia Hu, Ping Zhang, Hua Jiang, Jianghua Chen

**Affiliations:** ^1^ Kidney Disease Center the First Affiliated Hospital College of Medicine Zhejiang University Hangzhou China; ^2^ Key Laboratory of Kidney Disease Prevention and Control Technology Hangzhou China; ^3^ Institute of Nephrology Zhejiang University Hangzhou China; ^4^ State Key Laboratory for Diagnosis and Treatment of Infectious Diseases the First Affiliated Hospital College of Medicine Zhejiang University Hangzhou China

**Keywords:** acute kidney injury, chronic kidney disease, melatonin preconditioning, mesenchymal stem cells

## Abstract

Based on multiple studies in animal models, mesenchymal stem cell (MSC)‐based therapy appears to be an innovative intervention approach with tremendous potential for the management of kidney disease. However, the clinical therapeutic effects of MSCs in either acute kidney injury (AKI) or chronic kidney disease (CKD) are still under debate. Hurdles originate from the harsh microenvironment in vivo that decreases the cell survival rate, paracrine activity and migratory capacity of MSCs after transplantation, which are believed to be the main reasons for their limited effects in clinical applications. Melatonin is traditionally regarded as a circadian rhythm‐regulated neurohormone but in recent years has been found to exhibit antioxidant and anti‐inflammatory properties. Because inflammation, oxidative stress, thermal injury, and hypoxia are abnormally activated in kidney disease, application of melatonin preconditioning to optimize the MSC response to the hostile in vivo microenvironment before transplantation is of great importance. In this review, we discuss current knowledge concerning the beneficial effects of melatonin preconditioning in MSC‐based therapy for kidney disease. By summarizing the available information and discussing the underlying mechanisms, we aim to improve the therapeutic effects of MSC‐based therapy for kidney disease and accelerate translation to clinical application.

## 1 INTRODUCTION

Kidney disease is still a global public health problem in modern society and can be classified as acute kidney injury (AKI) or chronic kidney disease (CKD).^1^ AKI is characterized by an abrupt decline in glomerular filtration, while CKD is defined as a gradual loss of kidney function over 3 months.^2^, ^3^ The morbidity rate of kidney disease has increased rapidly in the last decade, and it is estimated that approximately 20% of patients will experience AKI during their hospitalization worldwide, while CKD affects approximately 13.6% of people in the United States.^4^, ^5^ AKI and CKD share an interactive pathophysiological process.^6^ Patients with AKI have an 8.8‐fold increased risk for developing CKD.^7^ Conversely, multiple studies have confirmed a significantly higher morbidity rate of AKI in patients with prior CKD.^8^, ^9^ For treatment, beyond the primary cause management, the therapeutic choice for AKI is still confined to supportive care or dialysis, which helps little in regenerating the injured kidneys.^10^ In CKD, although the application of renin‐angiotensin system inhibitors (RASIs) has shown benefits in delaying renal failure, treatments are unable to induce regression of glomerulosclerosis.^11^ The formation of fibrosis tissues in glomeruli and interstitial/tubules eventually leads to end‐stage renal disease (ESRD) in both of these diseases. In addition to renal involvement, renal function disorder will undoubtedly affect extrarenal organs given that the kidneys are important in maintaining body homoeostasis. Pathophysiological alterations following loss of renal function, such as dysregulation of extracellular volume and electrolytes and abnormal hormone secretion, can subsequently affect multiple extrarenal organs and induce a series of severe complications.^12^, ^13^ It has been reported that even mild renal injury was relevant to the development of cardiovascular system complications.^14^ The respiratory system, central nervous system, endocrine system and haematologic system, among others, may also be aggravated whether the abnormality is extensive or prolonged.^15^ The high incidence and poor prognosis of kidney disease are associated with significant economic costs, accounting for more than $10 billion for the treatment of AKI and over $80 billion to care for CKD patients annually.^16^, ^17^ Exploring new interventions to delay the progression of AKI and CKD is an urgent need.

**Figure 1:**
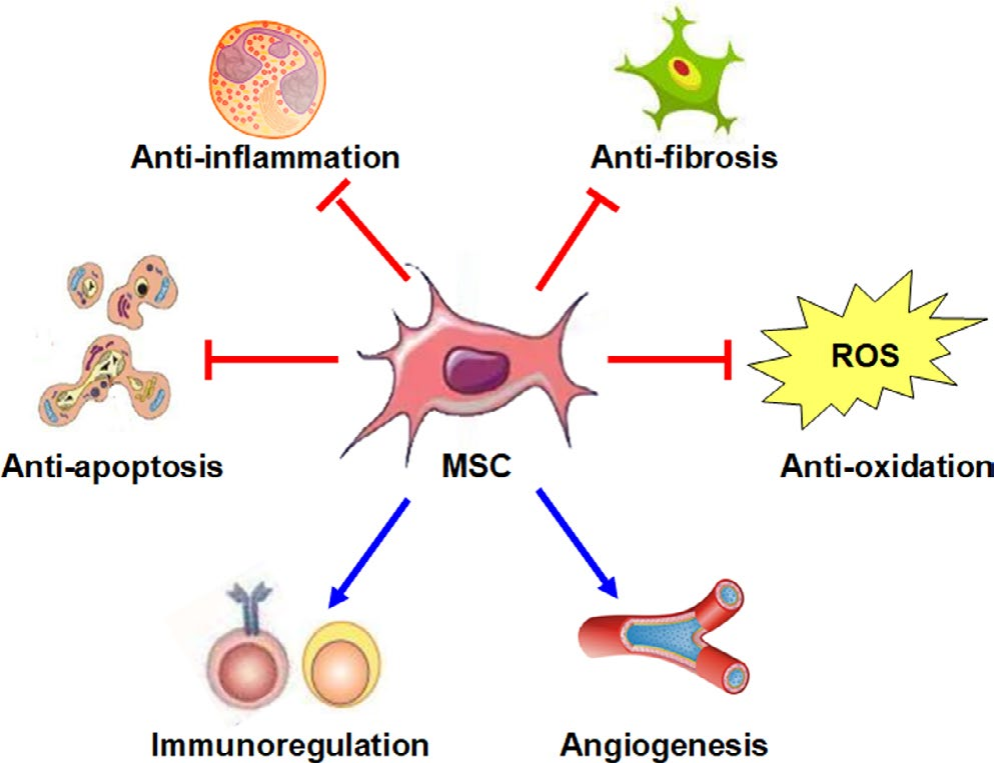
The principal mechanism by which MSCs exert their potential beneficial effects in kidney disease. The beneficial effects of MSC‐based therapy in kidney disease are reliant on the anti‐apoptotic, antioxidative, anti‐inflammatory, anti‐fibrotic, angiogenic and immunomodulatory properties of MSCs

In the last 10 years, we have witnessed an explosion in MSC‐based therapy for the management of AKI and CKD in multiple preclinical models. MSCs are fibroblast‐like multipotent cells that possess robust self‐renewal, regeneration, proliferation and multilineage differentiation ability.^18^, ^19^ Although the specific mechanisms underlying AKI and CKD are still not very clear, abnormalities in cell apoptosis or necrosis,^20^ inflammation,^21^, ^22^ immunoregulation,^23^ microvascular function,^24^, ^25^ oxidative stress injury^26^ and the expansion of fibroblasts/myofibroblasts^11^ are thought to play important roles during disease development. Numerous candidate agents targeting these abnormalities, such as Nec‐1,^27^ bindarit,^28^ OPN‐305,^29^ ephrinB2,^30^ SS‐31,^31^ bardoxolone methyl^32^ and pirfenidone,^33^ have shown promising therapeutic activity in some animal and clinical kidney disease models. While pharmacologic management is often confined to a single aspect of the highly complex pathophysiological process in AKI or CKD, MSCs are able to promote kidney repair through multiple mechanisms.^34^ A principal mechanism of these numerous MSC benefits resides in their paracrine/endocrine capacity. In general, it is considered that MSCs have the advantage of secreting a series of cytokines and growth factors that induce anti‐apoptotic,^35^ antioxidative,^36^ anti‐inflammatory,^37^ anti‐fibrotic,^38^ angiogenic^39^ and immunomodulatory^40^ activities (Figure 1). The development of MSC‐based regenerative medicine may bring hope to the billions of patients who suffer from kidney disease worldwide.

In this review, we will describe the current knowledge concerning MSC‐based therapy in kidney disease. We begin with the promising outcome of MSC‐based therapy in animal models, and the obstacles met in attempting to translate these outcomes into clinical application. Then, the underlying reasons are analysed, and different preconditioning strategies are presented. Finally, we will discuss the beneficial effects of melatonin preconditioning on protecting MSC function after transplantation and the underlying mechanism. By summarizing the current research, we hope to provide an integral and updated view of melatonin preconditioning in MSC‐based therapy for kidney disease.

## 2 SUCCESSFUL ATTEMPTS AT MSC‐BASED THERAPY IN ANIMAL MODELS AND HURDLES MET IN CLINICAL SETTINGS

The therapeutic effects of MSC‐based therapy in kidney disease have been confirmed in multiple animal models. The first study that indicated the renotropic property and tubular regenerative potential of MSCs was conducted in a mouse model of cisplatin‐induced AKI.^41^ This phenomenon was further verified in many subsequent studies that utilized many other AKI models, such as ischemia/reperfusion (I/R), sepsis and glycerol models.^42^ In the field of CKD, a meta‐analysis that included 71 articles also demonstrated that cell‐based therapy was valid for slowing the progression of CKD in preclinical settings.^43^ Based on these excellent results, some clinical trials were explored.

Currently, there are 18 completed or ongoing clinical trials associated with the application of MSCs in kidney disease according to ClinicalTrials.gov (Table 1). The first clinical trial in which the safety and efficacy of MSC therapy for AKI was evaluated was completed in 2013 (NCT00733876). MSCs were prophylactically transplanted into patients who were at high risk of developing AKI following cardiac surgery. Neither AKI nor any other adverse events occurred in the treatment group, while 20% of patients in the case‐controlled group developed AKI.^44^ Six autosomal dominant polycystic kidney disease (ADPKD) patients also presented tolerability to MSC therapy (NCT02166489). Moreover, a slowdown in the decline in the estimated glomerular filtration rate (eGFR) was also observed after MSC transplantation, suggesting the efficacy of this therapy. However, this trial was limited due to the lack of a control group.^45^ Saad et al transplanted MSCs via intra‐arterial injection into 14 patients with atherosclerotic renovascular disease (RVD). Patients in the intervention group showed better cortical perfusion and renal blood flow than those in the control group, demonstrating the therapeutic effects of the treatment (NCT01840540).^46^ Systemic lupus erythematosus (SLE) is another major cause of CKD, despite powerful immunosuppression regimens applied for its clinical management. To explore the safety and efficacy of MSC transplantation in refractory SLE patients, 13 patients underwent MSC therapy. At the end of the study, all patients presented tolerance to the regimen, together with decreased systemic lupus erythematosus disease activity index (SLEDAI) scores and proteinuria (NCT00698191).^47^ Similarly, a sequential multicenter clinical trial designed by the same team further confirmed that in patients with active and refractory SLE, MSC transplantation can significantly ameliorate disease activity, reduce proteinuria and improve renal function (NCT01741857).^48^

**Table 1:** Clinical trials of MSCs application in kidney disease

Condition	Aim of study	Enrolment	Phase	Status	Outcomes	ClinicalTrials.gov Identifier
AKI	To demonstrate the safety of allogeneic MSCs in patients who are at high risk of developing AKI following cardiac surgery	16	Phase I	Completed	Safe and efficient	NCT00733876
	To evaluate the safety and efficacy of allogeneic MSCs for the treatment of AKI after cardiac surgery	156	Phase II	Terminated	Safe but not efficient	NCT01602328
	To determine the safety and tolerability of extracorporeal MSCs in subjects with AKI receiving continuous renal replacement therapy	24	Phase I	Recruiting		NCT03015623
CKD	To provide confirmation of the safety of MSCs in patients with chronic renal failure due to ADPKD	6	Phase I	Completed	Safe and efficient	NCT02166489
	To develop a novel treatment via intra‐arterial MSC injection in atherosclerotic RVD patients	28	Phase I	Completed	Safe and efficient	NCT01840540
	To explore the safety and efficacy of MSC transplantation in refractory SLE	13	Phase I & II	Completed	Safe and efficient	NCT00698191
	To assess the safety and efficacy of MSCs in patients with active and refractory SLE	40	Phase I & II	Completed	Safe and efficient	NCT01741857
	To investigate the efficacy of MSCs for treatment of lupus nephritis	18	Phase II	Terminated	Safe but not efficient	NCT01539902
	To assess the safety, tolerability and therapeutic effects of mesenchymal precursor cells in patients with moderate to severe DN	30	Phase I & II	Completed	Safe but not efficient	NCT01843387
	To investigate the safety, feasibility, tolerability and efficacy of MSCs in subjects with progressive DN	48	Phase I & II	Recruiting		NCT02585622
	To determine whether intra‐renal delivery of MSCs would further enhance changes in single kidney blood flow and restoration of kidney function in human subjects with advanced RVD	42	Phase I	Not yet recruiting		NCT02266394
	To test the safety of intra‐parenchymal Wharton jelly‐derived MSC injection in the treatment of DN	20	Phase I & II	Not yet recruiting		NCT03288571
	To assess the safety, tolerability and efficacy of intra‐arterially delivered MSCs in patients with progressive DN	30	Phase I	Not yet recruiting		NCT03840343
	To investigate the treatment effects of MSCs in chronic renal failure patients	100	Not Applicable	Recruiting		NCT03321942
	To evaluate the safety and efficacy of MSCs for treatment of adults with active proliferative LN	36	Phase II	Not yet recruiting		NCT03673748
	To investigate the efficacy and safety of MSC transplantation in the treatment of LN compared with mycophenolate mofetil	230	Phase II	Not yet recruiting		NCT03580291
	To demonstrate the safety and efficacy of MSCs in patients with SLE and LN	30	Not Applicable	Not yet recruiting		NCT03458156
	To assess the safety and tolerability of MSCs (CS20AT04) in subjects with LN	6	Phase I	Not yet recruiting		NCT03174587

Abbreviations: ADPKD, autosomal dominant polycystic kidney disease; AKI, acute kidney injury; CKD, chronic kidney disease; DN, diabetic nephropathy; DN, diabetic nephropathy; LN, lupus nephritis; MSCs, mesenchymal stem cells; RVD, renal vascular disease; SLE, systemic lupus erythematosus.

In addition to the above‐mentioned exciting results, contradictory results have been reported in other studies. A phase II, randomized, multicenter trial was terminated due to the uncertain therapeutic effects in patients with postcardiac surgical AKI (NCT01602328).^49^ Packham et al aimed to assess the safety, tolerability and therapeutic effects of mesenchymal precursor cells in patients with moderate to severe diabetic nephropathy, which is a major cause of CKD. After follow‐up for 12 weeks, the measured parameters, including serum creatinine, creatinine clearance, albumin‐creatinine ratio, protein‐creatinine ratio, HbA1c and blood pressure, were comparable in both groups (NCT01843387).^50^ In the aspect of lupus nephritis (LN), the two studies mentioned above were both observational studies, which might provide insufficiently strong evidence. In a randomized double‐blind, placebo‐controlled trial that was published in 2017, MSC therapy did not show better therapeutic effects than the placebo (NCT01539902).^51^ These contradictory results confuse physician when making clinical decisions. However, there are still approximately ten registered or ongoing trials in this field. We are looking forward to the results of these studies, which may lead to a clear conclusion about the clinical effects of MSC‐based therapy for kidney disease.

Why is there still a huge gap between experimental evidence and clinical applications? One major reason for the limited clinical effects of MSCs in kidney disease is the reduced cell function after transplantation.^52^ As mentioned above, the beneficial effects of MSCs are related to their paracrine/endocrine activity and largely rely on the number of MSCs at the site and the concentration of secreted cytokines/growth factors around the damaged tissues.^53^ However, different from the appropriate culture conditions ex vivo, MSCs face an undesirable microenvironment after transplantation that may induce early cell death and loss of function. In one study, more than 80%–90% of grafted cells died within 72 hours.^54^ Data from Freyman et al also showed that only approximately 3% of administered MSCs remained alive at 14 days after injection.^55^ Moreover, decreased angiogenesis capacity, defects in migration activity and accelerated cellular senescence were also observed in MSCs after exposure to uremic toxins during AKI or CKD.^56^, ^57^ For the secretory activity, Idziak et al and Klinkhammer et al noticed that after exposure to uremic toxins, the secretomes of MSCs were largely altered. Up‐regulated secretion of IL‐8 and PDGF and decreased expression of VEGF converted MSCs into a more inflammatory, more fibrotic and less regenerative phenotype, which had a profound consequence on its anti‐inflammatory, anti‐fibrotic, angiogenic and immunomodulatory functions.^58^, ^59^ Finding a proper preconditioning strategy to optimize MSCs and enhance their survival, paracrine effects and migratory ability before transplantation is of great importance.^60^

## 3 MELATONIN PRECONDITIONING AND MSC‐BASED THERAPY FOR KIDNEY DISEASE

**Figure 2:**
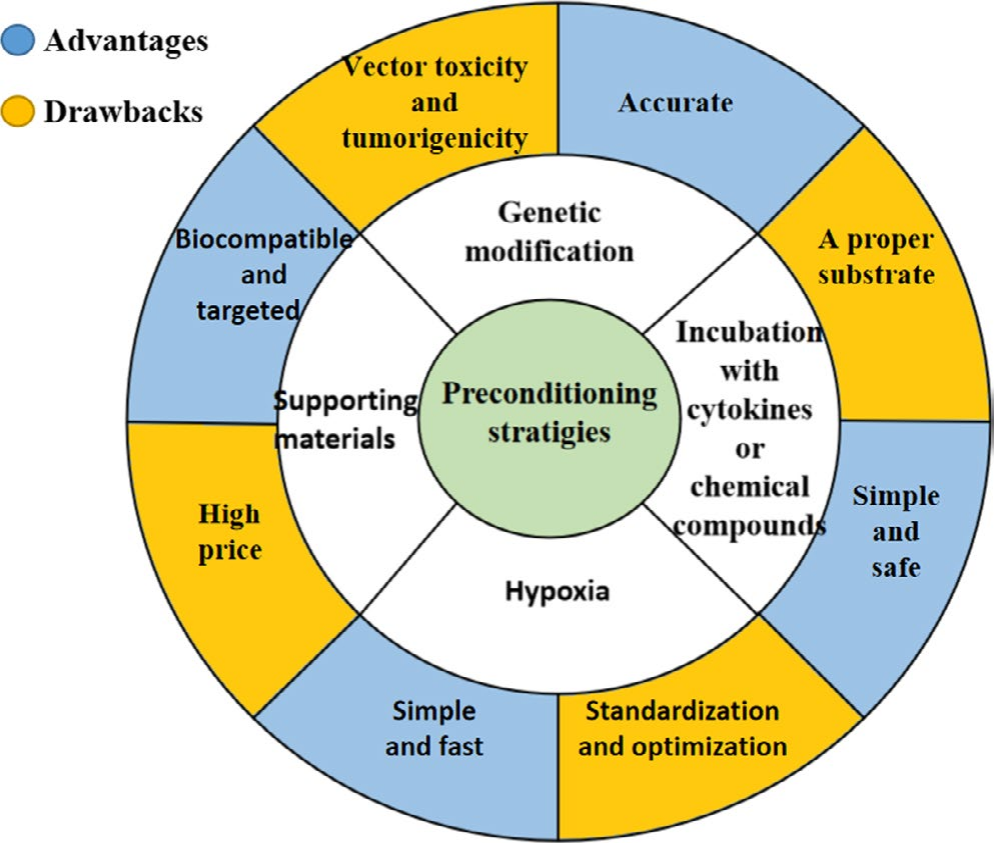
Advantages and drawbacks of different preconditioning strategies. Incubation with cytokines or chemical compounds, hypoxia preconditioning, application of supporting materials and genetic modification are currently the four main preconditioning strategies. These four preconditioning strategies have unique advantages and drawbacks. Currently, it is difficult to say one strategy is greater than another

Multiple preconditioning strategies have been developed in recent years. Generally, these strategies can be sorted into four categories: incubation with cytokines or chemical compounds, hypoxia preconditioning, application of supporting materials and genetic modification. Different preconditioning strategies have unique advantages and drawbacks (Figure 2). Hypoxia preconditioning is simple and fast but faces the issues of standardization and optimization.^61^ Genetic modification is a more accurate approach compared with other preconditioning strategies but presents a risk of vector toxicity and tumorigenicity.^62^ The advantages of application with supporting materials are greater biocompatibility and targeting; however, the current price of these newly developed materials is still high, which may restrict their application in the clinic.^63^ In addition to the above‐mentioned methods, incubation of MSCs with cytokines or chemical compounds is another preconditioning strategy that has long been explored. The simple operation and safety guarantee of this technology make it a promising preconditioning strategy for clinical application, especially when the cells are incubated with certain physiological hormones.^52^ However, the key challenge of this technique is to find a proper substrate.

Melatonin is a neurohormone that is primarily secreted by the pineal gland. The physiological role of melatonin is a key regulatory molecule in circadian rhythms.^64^ A low blood level of melatonin during the daytime and an increased level at night‐time guarantees a sleep‐wake cycle in mammals.^65^ In addition to its traditional role, in recent years, melatonin has been found to take part in many other pathophysiologic processes. By possessing antioxidant and anti‐inflammatory properties,^66^, ^67^ melatonin was found to be a potent free radical scavenger and present protective effects in multiple dysfunctional organs, including the kidneys.^68^, ^69^ Immune function during the course of prostate cancer therapy has also been demonstrated.^70^ Based on the fact that inflammation, oxidative stress, thermal injury and hypoxia are four main factors that cause the dysfunction of injected MSCs under disease conditions, preconditioning with melatonin may become a wonderful strategy.^71^ In addition, melatonin is currently applied as a dietary complement and shows little risk of genetic mutation, tumorigenicity or other major side effects.^72^, ^73^

**Figure 3:**
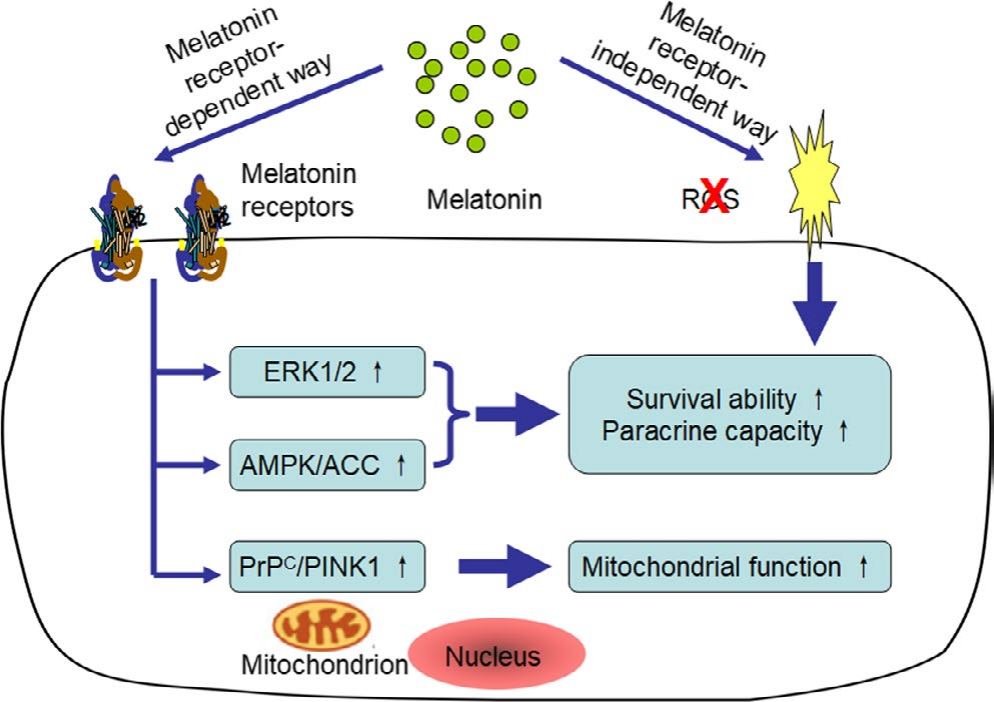
The mechanism underlying the protective effects of melatonin on MSCs. Melatonin preconditioning exerts beneficial effects on MSCs through receptor‐dependent and receptor‐independent pathways. By eliminating ROS and up‐regulating the ERK1/2, AMPK/ACC, and PrP^C^/PINK1 signaling pathways, this method can facilitate cell survival and promote paracrine activity and mitochondrial function

Incubation of MSCs with melatonin prior to transplantation has been confirmed to be able to induce an enhanced therapeutic outcome in multiple animal models, including myocardial infarction, cerebral ischemia and limb ischemia models.^74^, ^75^, ^76^ In a broad sense, it was considered that melatonin itself could efficiently serve as an antioxidant and protect MSCs from oxidation injury by biologically eliminating free radicals.^66^ In addition to receptor‐independent pathways, the melatonin receptors MT1 and MT2 have also been found to be highly expressed on the surface of MSCs, indicating that melatonin may regulate the fate of MSCs in a receptor‐dependent manner.^77^, ^78^ Moreover, enhanced PrP^C^‐dependent mitochondrial function,^79^ Erk1/2 overexpression^80^ and up‐regulated phosphorylation of AMPK pathway proteins were observed after melatonin preconditioning.^81^ Melatonin preconditioning can rely on various mechanisms to protect injected MSCs against premature senescence or early apoptosis after transplantation and definitely exaggerate their therapeutic effects in diseased tissues (Figure 3). However, whether these benefits still exist in kidney disease is unclear. In the following section, we will discuss this question (Table 2).

**Table 2:** Studies demonstrating the beneficial effects of melatonin preconditioning in MSC‐based therapy for kidney disease

Condition	References	Year	Animal	Model	Stem cells source	Renal outcomes	MSCs outcomes
AKI	Chen^83^	2014	Rats	Sepsis‐AKI	A‐AMSCs	↓Inflammatory; ↑Antioxidants; ↓ROS; ↓Apoptosis; ↓Fibrosis; ↓Kidney injury score; ↑Renal function	Not mentioned
	Zhao^84^	2015	HK‐2 cells	Cisplatin‐AKI	AMSCs	↑Proliferation; ↑Migration; ↑Prosurvival and anti‐apoptotic ability	↑Proliferation; ↑Prosurvival, anti‐apoptotic and antioxidative ability;
	Mias^53^	2008	Rats	I/R‐AKI	BMMSCs	↑Angiogenesis; ↑Proliferation; ↓Apoptosis; ↑Renal function	↓Apoptosis; ↑Survival rate; ↑Paracrine ability (b‐FGF, HGF);
CKD	Saberi^71^	2019	Rats	UUO	BMMSCs	↓TGF‐β and TNF‐α; ↓α‐SMA; ↑E‐cadherin; ↓Fibrotic areas	↑Survival rate; ↑Migratory activity
	Rashed^87^	2018	Rats	DN	MSCs	↓TGF‐β; ↑SOD‐1; ↑Beclin‐1; ↑Renal function	↑Proliferation

Abbreviations: A‐AMSCs, apoptotic adipose‐derived MSCs; AKI, acute kidney injury; AMSCs, adipose‐derived MSCs; b‐FGF, basic fibroblast growth factor; BMMSCs, bone marrow‐derived mesenchymal stem cells; Cisplatin‐AKI, cisplatin‐induced AKI; CKD, chronic kidney disease; DN, diabetic nephropathy; HGF, hepatocyte growth factor; I/R, ischemia/reperfusion; I/R‐AKI, I/R induced AKI; MSCs, mesenchymal stem cells; ROS, reactive oxygen species; Sepsis‐AKI, sepsis induced AKI; SOD‐1, superoxide dismutase‐1; TGF‐β, tumour growth factor β; TNF‐α, tumour necrosis factor‐ α; UUO, unilateral ureteral obstruction; α‐SMA, α‐smooth muscle actin.

### 3.1 Application of MSCs with melatonin preconditioning to treat AKI

Sepsis‐induced AKI (sepsis‐AKI) is a major subtype of AKI with high morbidity and mortality.^82^ To assess whether MSCs treated with melatonin can exert additional benefits in attenuating sepsis‐AKI, Chen et al conducted a series of experiments that aimed to compare a combination of melatonin and apoptotic adipose‐derived MSCs (A‐ADMSCs) with A‐ADMSCs alone in a rat model of sepsis. After cecal‐ligation and puncture (CLP), the combined treatment presented better therapeutic effects in the aspects of anti‐inflammation, antioxidization, anti‐apoptosis, and anti‐fibrosis activity and the circulating level of creatinine. Haematoxylin and eosin (HE) staining also confirmed less kidney injury in the combined treatment group.^83^ To gain further sight into the mechanisms underlying the protective effects of melatonin on MSCs, Zhao et al evaluated the action of melatonin on adipose‐derived MSCs (AMSCs) ex vivo. Melatonin pretreatment significantly enhanced the proliferation of AMSCs according to MTT assays. Moreover, higher expression of P‐Erk1/2, P‐Akt, superoxide dismutase‐1 (SOD‐1) and haeme oxygenase‐1 (HO‐1) was observed, indicating better prosurvival, antiapoptotic and antioxidative capacity of AMSCs after exposure to melatonin. Furthermore, their study also demonstrated the superiority of conditioned medium from melatonin‐pretreated AMSCs in enhancing the proliferative, migratory, prosurvival and antiapoptotic abilities of human HK‐2 cells exposed to cisplatin.^84^ To assess whether these protective mechanisms are still effective in vivo, Mias et al transplanted bone marrow‐derived mesenchymal stem cells (BMMSCs) preconditioned with melatonin into ischemia/reperfusion‐induced AKI (I/R‐AKI) rats. More surviving grafted MSCs, overstimulation of angiogenesis and proliferation in renal cells and more rapid renal function recovery were observed in the preconditioning group, indicating a potential role of melatonin in protecting transplanted MSCs in the in vivo microenvironment. In vitro analysis demonstrated a higher level of catalase and SOD‐1 expression and overexpression of basic fibroblast growth factor (bFGF) and hepatocyte growth factor (HGF) in the preconditioning group. However, all these beneficial effects were reversed by the melatonin receptor antagonist luzindole, suggesting that melatonin is able to enhance the intrinsic prosurvival and paracrine abilities of MSCs through its receptors.^53^

### 3.2 Application of MSCs with melatonin preconditioning to treat CKD

To date, only two studies have described the beneficial effects of melatonin on MSC‐based therapy in the field of CKD. Saberi et al pretreated MSCs with melatonin and then transplanted them into unilateral ureteral obstruction (UUO) rats. They found that pretreated MSCs could, on the one hand, decrease the expression of transforming growth factor‐β (TGF‐β), tumour necrosis factor‐α (TNF‐α) and α‐smooth muscle actin (α‐SMA) and, on the other hand, increase the expression of E‐cadherin compared with the control group, which ultimately induced fewer fibrotic areas in renal tissues. In addition, more engrafted and surviving pretreated MSCs were observed in the injured kidneys, suggesting a better survival and migratory activity of MSCs after melatonin incubation.^71^

Diabetic nephropathy (DN) is a major cause of CKD and shows increasing morbidity worldwide.^85^ MSC‐based therapy could become a promising therapeutic strategy for DN.^86^ However, local oxidative stress and the inflammatory state might largely impact the migratory and survival capacities of MSCs in diabetic tissues.^57^ Rashed et al preincubated MSCs with melatonin and found that melatonin pretreatment significantly enhanced the proliferation of MSCs in vitro. Then, they injected melatonin‐preincubated MSCs into rats with DN. Compared with the control group, the rats that received preincubated MSC therapy presented better kidney function and greater amelioration of the underlying pathogenic factors. This study verified the superiority of ex vivo melatonin treatment, which acts as a preconditioning agent to enhance the efficiency of MSCs therapy in DN.^87^

## 4 CONCLUSION AND FUTURE PERSPECTIVES

Given the increasing prevalence of AKI and CKD worldwide, continued breakthroughs in the field of MSC‐based therapy have a large potential impact and social benefits. Numerous clinical trials have confirmed the tolerability and safety of MSC treatment in kidney disease. However, some concerns regarding their unproven therapeutic effects in clinical applications remain due to their reduced cell function after transplantation. In this review, we summarized the currently available studies in which melatonin was used as a preconditioning substrate to enhance the therapeutic effects of MSCs in kidney disease. These studies definitively demonstrate the superiority of this strategy both in vivo and ex vivo.

In terms of mechanism, the benefits of melatonin include but are not limited to its antioxidative and anti‐inflammation effects. Current evidence indicates that melatonin preconditioning can also significantly enhance the proliferative, prosurvival, paracrine secretion and migratory abilities of MSCs after transplantation. One major advantage of this strategy over other preconditioning methods is the safety issue; melatonin can be applied as a dietary complement. However, at the moment, there are no available articles comparing the ability of melatonin with that of other preconditioning methods to enhance the capacity of MSCs. Whether melatonin preconditioning is superior to other preconditioning strategies should be explored in future studies.

We believe that summarizing the related articles and clarifying the underlying mechanism will shed light on the protective role of melatonin preconditioning in MSC‐based therapy for kidney disease. However, robust clinical evidence concerning this strategy in clinical settings is still lacking. We look forward to a promising future of MSC‐based therapy for kidney disease and call for more clinical trials in this field.

## CONFLICT OF INTEREST

The authors confirm that there are no conflicts of interest.

## AUTHOR'S CONTRIBUTION

LF Zhao and JH Chen contributed to the conception of the manuscript. LF Zhao and CX Hu were responsible for the literature review. LF Zhao, P Zhang and H Jiang drafted and revised the manuscript. All authors read and approved the final manuscript.
